# Oscillatory Shear Stress Induces Oxidative Stress via TLR4 Activation in Endothelial Cells

**DOI:** 10.1155/2019/7162976

**Published:** 2019-06-17

**Authors:** Zhimei Wang, Feng Wang, Xiangquan Kong, Xiaofei Gao, Yue Gu, Junjie Zhang

**Affiliations:** Department of Cardiology, Nanjing First Hospital, Nanjing Medical University, Nanjing, China

## Abstract

**Background:**

Oscillatory shear stress (OSS) disrupts endothelial homeostasis and promotes oxidative stress, which can lead to atherosclerosis. In atherosclerotic lesions, Toll-like receptor 4 (TLR4) is highly expressed. However, the molecular mechanism by which TLR4 modulates oxidative changes and the cell signaling transudation upon OSS is yet to be determined.

**Methods and Results:**

Carotid artery constriction (CAC) surgery and a parallel-plate flow chamber were used to modulate shear stress. The results showed that OSS significantly increased the oxidative burden, and this was partly due to TLR4 activation. OSS activated NOX2 and had no significant influence to NOX1 or NOX4 in endothelial cells (ECs). OSS phosphorylated caveolin-1, promoted its binding with endothelial nitric oxide synthase (eNOS), and resulted in deactivation of eNOS. TLR4 inhibition restored levels of nitric oxide (NO) and superoxide dismutase (SOD) in OSS-exposed cells.

**Conclusion:**

TLR4 modulates OSS-induced oxidative stress by activating NOX2 and suppressing eNOS.

## 1. Introduction

Arterial endothelium homeostasis is associated with the distribution of shear stress, a dragging force generated by blood flow which has a profound effect on endothelial function [1]. Endothelial cells (ECs), which comprise the inner surface of the vessels, are exposed to various flow patterns such as laminar shear stress and oscillatory shear stress (OSS) directly [1, 2]. OSS prefers to appear at the curvatures, bifurcations, and branches in the artery, where the fluid mechanical environment is distinct from the straight sections of the vessel wall [3]. Cells in regions undergoing OSS are characterized by accumulated reactive oxygen species (ROS), decreased nitric oxide (NO) bioavailability, and prevailed inflammation [4]. Endothelial dysfunction is the initial factor leading to atherosclerosis lesions, where an accumulation of Toll-like receptors (TLRs) has been found [5, 6].

TLRs, pattern recognition receptors, are part of the innate immune system and respond to pathogenic factors or cellular damage to elicit an effective defense [5, 7]. Recently, a growing body of evidence has elucidated their role in regulating the inflammatory response and maintaining endothelial homeostasis [5]. TLR4, the first identified TLR, has also been implicated in the development and progression of cardiovascular disease [8, 9]. Several studies have reported a role of TLR4 in promoting ECs proliferation and neointima formation [10]. Lu et al. identified a high expression of TLR4 in endothelial cells and macrophages in atherosclerotic plaques [11]. Our previous genome analysis indicated that TLR4 was the most differentially expressed mRNA in sheared cells compared with static cultured cells [12]. We have also shown that acute exposure to shear stress results in extensively oxidative damage in ECs [13, 14]. However, the effect of TLR4 activation in sheared cells and the related mechanism remain unclear. In the present study, we demonstrate that OSS activates TLR4, causing downstream effects on NOX2 and eNOS that result in oxidative damage.

## 2. Material and Methods

### 2.1. Cell Culture

Human umbilical vein endothelial cells were obtained from the Type Culture Collection of the Chinese Academy of Sciences (Shanghai, China). Cells were maintained in Dulbecco's Modified Eagle's Medium culture medium supplemented with 10% fetal bovine serum and cultured at 37°C in a humidified incubator with 5% CO_2_. When grown to confluence, cells were trypsinized, harvested, resuspended, and seeded to a 0.1% gelatin-coated glass. After adherence, cells were used for shear stress study.

### 2.2. Parallel-Plate Flow Chamber Study

A parallel-plate flow chamber that exerts continuous flow was made by sandwiching a silicon gasket between two stainless steel plates with a cover slip sink in the base plate. The chamber and all parts of the circuit were sterilized by steam autoclaving, before a glass plate containing monolayer cells was placed into the flow chamber. Shear stress is calculated as *τ* = 6∗*Q*∗*μ*/(*w*∗*h*^2^), in which *τ* is the target shear stress acting tangentially on the cells, *Q* is the flow rate, *μ* is the viscosity of the perfusate, and *w* and *h* refer to the width and height of the flow chamber. In this experiment, the wall shear stress used was 4 dynes/cm^2^, the viscosity of the medium was 0.009 g/cm∗s, and the width and height of the chamber were 28 mm and 440 *μ*m, respectively.

### 2.3. Carotid Artery Constriction

**Figure 1:**
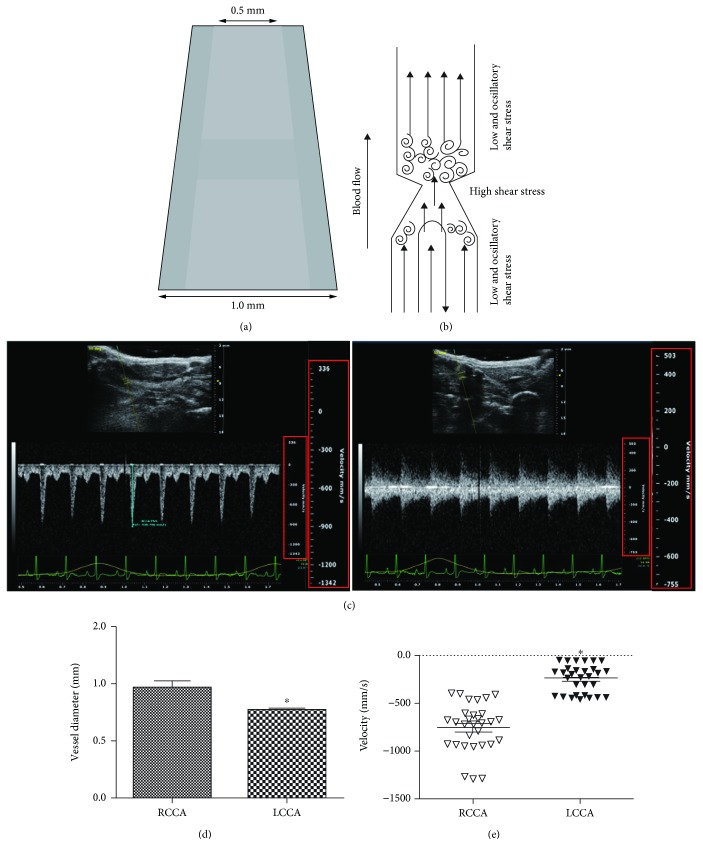
Flow change of the left common carotid artery (LCCA) and right common carotid artery (RCCA) after CAC surgery. (a) A silicon model was used in CAC surgery. (b) A schematic of flow change after CAC surgery. (c) LCCA was constricted and the artery ultrasound detected reverse blood flow. (d) CAC surgery decreased vessel diameter and (e) flow velocity of LCCA. ^∗^*p* < 0.05 versus the RCCA, *n* = 30.

Carotid artery constriction (CAC) was conducted to modify flow pattern. Briefly, 6-8 weeks old rats were anesthetized with pentobarbital (20 mg/kg, ip) and subjected to CAC surgery using a modified cast, which was cone-shaped and made with silicone (Figure 1(a)). In the sham group, the right carotid was exposed but was left unconstricted. Blood velocity was measured using a Doppler ultrasound at 7 days postsurgery, the rats were subsequently sacrificed, and their carotids were removed for further study. The experimental and animal surgery procedures were performed in accordance with the National Institutes of Health Guide for the Care and Use of Laboratory Animals and approved by the animal ethics board of Nanjing Medical University.

### 2.4. Tissue Section of Human Coronary Artery

Human coronary arteries were collected from patients undergoing heart transplant surgery that has been approved by the Institutional Review Board of Nanjing First Hospital. We separated the left main coronary artery, left descending artery, and left circumflex artery carefully. Arteries were divided into bifurcation and nonbifurcation groups. After that, tissues were embedded with OCT and cut into slices which were then stained with primary antibodies followed with another incubation of fluorescence-conjugated secondary antibodies. Images were obtained using a confocal microscope (ZEISS, German).

### 2.5. Transient Transfection with siRNA

Human NOX2 siRNA, human TLR4 siRNA, and a negative control siRNA with a scrambled sequence were purchased from GenePharma (Shanghai, China). The nucleotide sequences of siRNA against NOX2 and TLR4 were as follows: siNOX2, 5 ′-GGGUUUAUGAUAUUCCACCUAAGUU-3 ′ (sense) and 5 ′-AACUUAGGUGGAAUAUCAUAAACCC-3 ′ (antisense); siTLR4, 5 ′-GCUGAUGCCGCUGAUGCCATT-3 ′ (sense) and 5 ′-UGGCAUCAGCGGCAUCAGCTT-3 ′ (antisense). Cells were transfected at 50% confluence using lipofectamine 3000 reagent according to the manufacturer's instructions, with a final siRNA concentration of 50 nmol/L. After incubation for 6 h, the cells were fed with growth medium, and after 48 h, the cells were exposed to OSS.

### 2.6. Coimmunoprecipitation

Following flow treatment, ECs were lysed and incubated with caveolin-1 overnight at 4°C with gentle shaking. Following further incubation with protein A+G for another 4 h, the precipitate was washed five times with RIPA at 1,000 g for 5 min and then resuspended in SDS-PAGE loading buffer. After being heated at 99°C for 5 min, samples were separated by SDS-PAGE and transferred to a PVDF membrane to detect total eNOS. Primary antibodies against NOX2 and caveolin-1 were from Abcam. (Cambridge, UK). Antibodies against p47 and eNOS were purchased from Cell Signaling Technology (Massachusetts, USA) and R&D Systems Inc. (Minneapolis, USA), respectively. Protein A+G was purchased from Millipore (Massachusetts, USA).

### 2.7. Western Blot Analysis

Monolayer cells grown on a glass plate were subjected to OSS for different lengths of time (0, 20, 40, 80, and 120 min); cells were then lysed in a cocktail of RIPA, proteinase inhibitor, and phosphatase inhibitor. After that, the lysates were centrifuged at 12,000 g for 15 min at 4°C, and the supernatant was collected for concentration determination and protein detection. Protein concentration was quantified using a bicinchoninic acid protein assay according to the manufacturer's instructions (KeyGen Biotech, Nanjing, China). In total, 60 *μ*g protein was separated by SDS-PAGE and transferred to a polyvinylidene difluoride membrane, which was then incubated overnight at 4°C with primary antibody. Following this, the membrane was washed and incubated with horseradish peroxidase-conjugated secondary antibody for 2 h at room temperature and developed using an enhanced chemiluminescence kit. Bands were analyzed using ImageJ software. Primary antibodies against GAPDH, caveolin-1, and p47 were obtained from Cell Signaling Technology (Massachusetts, USA). Primary antibodies against NOX1, NOX2, and NOX4 were from Abcam (Cambridge, UK). Primary antibody against eNOS was from R&D Systems Inc. (Minneapolis, USA). Antibody against TLR4 was purchased from Santa Cruz (Dallas, USA). Horseradish peroxidase-coupled secondary antibody was purchased from Santa Cruz (Dallas, USA).

### 2.8. Confocal Microscopy

Cells or tissues mounted on glass slides were gently washed twice with PBS, followed by fixation with 4% paraformaldehyde for 20 min. After permeabilization with 0.1% TritonX-100 for 5 min, they were incubated with 3% BSA for 1 hour at room temperature and then with primary antibodies overnight at 4°C. After that, they were incubated with Fluor 488- or Fluor 555-conjugated secondary antibodies for 2 hours at room temperature, and DAPI was used to counter stain nuclei. Finally, images were obtained using a confocal microscope (ZEISS, German). Primary antibodies against NOX2, caveolin-1, and CD31 were from Abcam (Cambridge, UK). Antibody against eNOS was obtained from R&D Systems Inc. (Minneapolis, USA). Antibody against TLR4 was purchased from Santa Cruz (Dallas, USA). Fluor 488- or 555-conjugated secondary antibodies were from Servicebio (Wuhan, China).

### 2.9. Measurement of ROS Accumulation

ROS level was detected through either chemiluminescence or flow cytometry. For chemiluminescence assay, cells following treatment (static culture, OSS 30 min, LPS, and TAK-242+OSS 30 min) were gently washed twice with ice-cold PBS, incubated with ROS Fluorescent Probe-DHE (5 *μ*mol/L, Beyotime) at 37°C for 20 min under light-protected conditions and stained with DAPI. Images were obtained with a confocal microscope (ZEISS, German). For flow cytometry analysis, cells that are treated with apocynin, allopurinol, tempol, and rotenone were applied with OSS; after that, cells were trypsinized, resuspended, and incubated with ROS Fluorescent Probe-DHE (5 *μ*mol/L, Beyotime) for analysis. At least 10,000 events were analyzed, and the intensity of fluorescence was determined using the PE-A channel. Data were analyzed using FlowJo V10.3.0 software. LPS was purchased from Sigma-Aldrich (St. Louis, USA). TAK-242 was from MedChemExpress (Shanghai, China). Apocynin, allopurinol, tempol, and rotenone were purchased from Selleck (Houston, USA).

### 2.10. Assessment of NO Bioavailability

NO was analyzed to confirm the protection of TLR4 inhibition on OSS-treated cells. Cells subjected to OSS for 30 min with or without TLR4 inhibition were lysed, scraped, and centrifuged. Subsequently, the supernatant was incubated with Griess Reagents, and the mixture was examined at 540 nm according to the manufacturer's instructions (KeyGen Biotech Co. Ltd, Nanjing, China).

### 2.11. Determinant of SOD Activity

SOD was tested to confirm the antioxidant effect of TLR4 inhibition on OSS-treated cells. In brief, sheared cells with TLR4 inhibited or not were lysed, and the lysates were centrifuged. After that, supernatant was collected and was incubated with WST-8/enzyme working solution and SOD detected buffer solution for 30 min at 37°C. At last, the mixture was assayed at 450 nm. This detection was conducted according to the manufacturer's instructions (Beyotime, Nanjing, China).

### 2.12. Data Analysis and Statistics

Quantitative data were presented as means ± SEM. Student's *t*-test was used to analyze data between two groups, and one-way analysis was utilized to compare data from more than two groups. Statistical significance was assumed as *p* < 0.05, and all analyses were performed with SPSS version 19.0 (Chicago, USA).

## 3. Results

### 3.1. CAC Induces Flow Change in Rat Carotid Arteries

A silicone-made, cone-shaped cast was used to modify shear stress in the left common carotid artery (LCCA) of rats (Figure 1(a)). After placement of the constricted cast, the downstream region will be exposed to oscillations in shear stress. The tapering region, where the vessel diameter decreases from 1 mm to 0.5 mm, induces a gradual increase in shear stress. In the upstream region of the cast, blood velocity reduced and shear stress decreased (Figure 1(b)). Blood flow was analyzed by a Doppler ultrasound at 7 days postsurgery. Compared to sham, the constricted arteries exhibited reverse flow at the proximal part of the cast, which suggested an oscillatory flow pattern. In addition, the modified vessels showed narrowing and reduced maximal blood velocity (Figures 1(c)–1(e)).

### 3.2. OSS Activates TLR4 Both In Vivo and In Vitro

**Figure 2:**
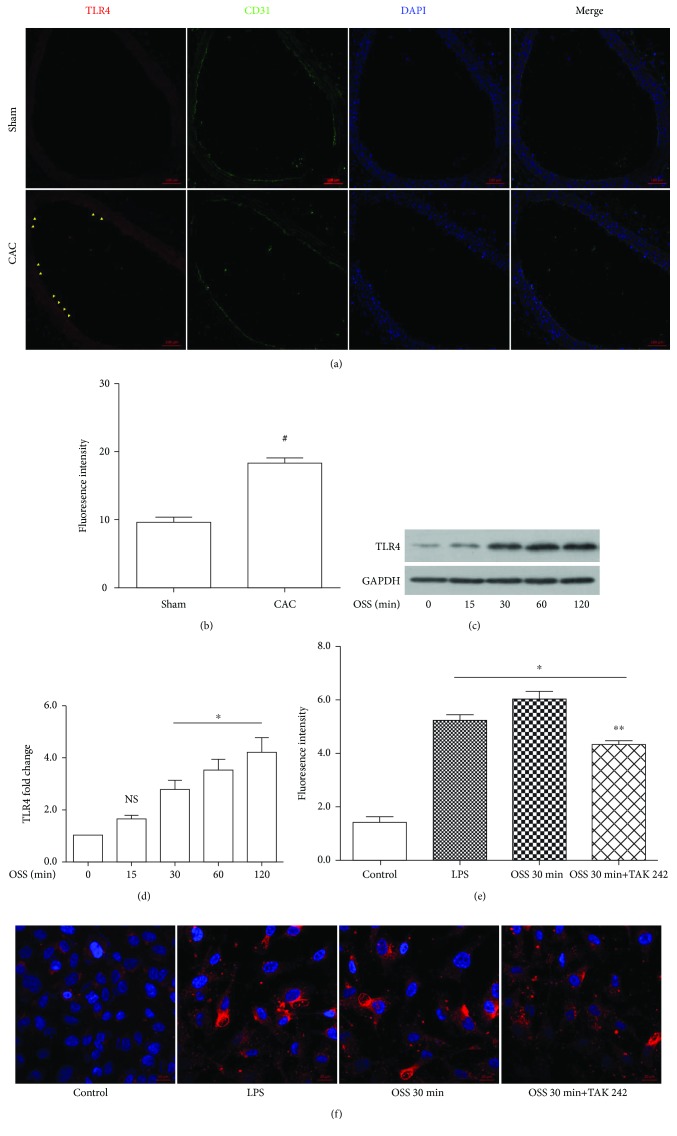
OSS activates TLR4 signaling. (a) TLR4 expression in carotid arteries and CD31 was used to identify endothelium. (b) Analysis for intensity of florescence. (c) Representative immunoblots for TLR4 in OSS-treated cells, the level of proteins was normalized by GAPDH. (d) Bar diagram showing quantitative data of immunoblots. (e) Analysis for mean intensity of florescence. (f) Representative images of ROS-positive cells in static-cultured cells, LPS-treated cells, OSS 30 min-treated cells with or without the preincubation of TAK-242 (1 *μ*mmol/L). ^#^*p* < 0.05 versus sham, *n* = 6; ^∗^*p* < 0.05 versus control group, ^∗∗^*p* < 0.05 versus OSS 30 min, *n* = 3.

We have previously conducted GeneChip analysis to generate an mRNA expression profile in shear stress-treated cells and found that TLR4 was one of the most differentially expressed genes. In this study, we assayed the TLR4 expression in carotid arteries and found increased levels of TLR4 in the OSS exposure regions (Figures 2(a) and 2(b)). In vitro study, a parallel-plate flow chamber was used to mimic flow changes. In response to OSS, TLR4 was activated at 30 min and sustained until 120 min (Figures 2(c) and 2(d)). Oxidative changes appeared immediately after the application of shear stress; hence, we compared oxidative states in static-cultured cells, LPS-treated cells, and OSS-treated cells with or without TAK-242 (1 *μ*mmol/L) preincubation. Chemiluminescence analysis reflected a comparable ROS level in cells treated with OSS or LPS; TAK-242 restored OSS-induced oxidative burden (Figures 2(e) and 2(f)). Taken together, the above results suggested that the oxidative burden trigged by OSS was at least in part due to TLR4 activation.

### 3.3. OSS Activates NADPH Oxidase

**Figure 3:**
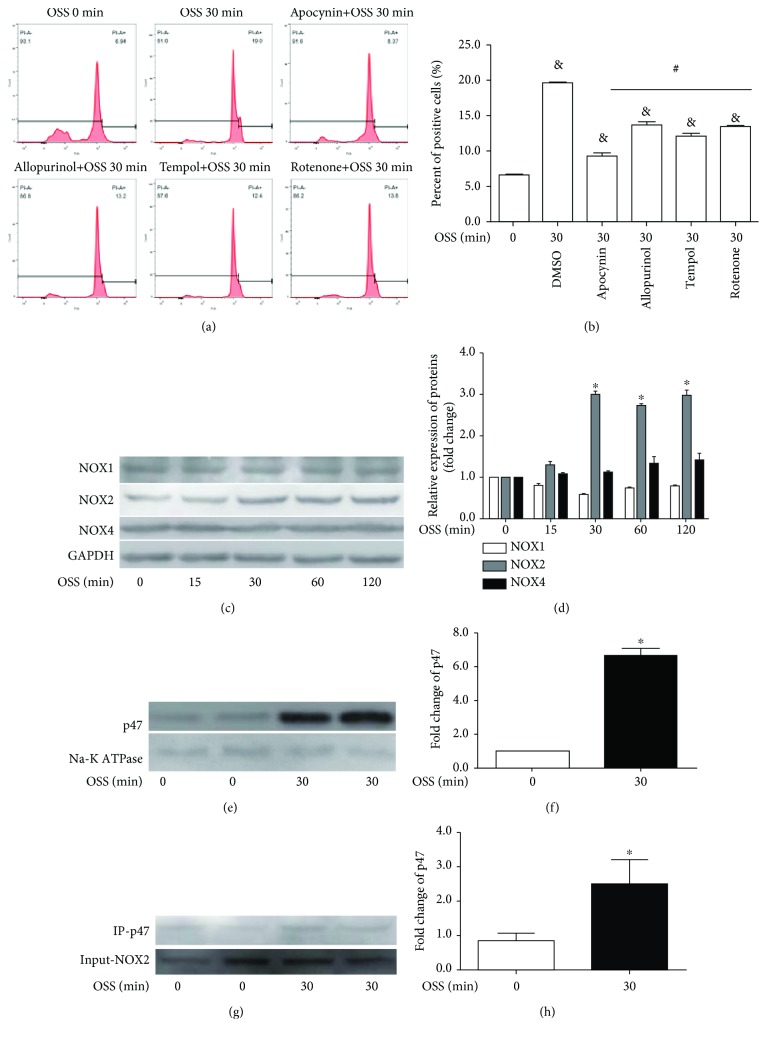
OSS activates NOX2. (a) Antioxidant effect of apocynin (10 *μ*mmol/L), allopurinol (1 mmol/L), tempol (1 mmol/L), and rotenone (100 *μ*mmol/L) against OSS-induced oxidative stress. (b) Percentage of ROS-positive cells. (c) Representative immunoblots for NOX1, NOX2, and NOX4 in OSS-treated cells. (d) Densitometry analysis of immunoblots for NOX1, NOX2, and NOX4. (e) Immunoblots of membrane p47. (f) Densitometry analysis of immunoblot for p47. (g) Coimmunoprecipitation of p47 and NOX2 in whole cell lysate. (h) Bar diagram showing quantitative analysis of p47. ^&^*p* < 0.05 versus OSS 0 min, ^#^*p* < 0.05 versus OSS 30 min, *n* = 6; ^∗^*p* < 0.05 versus OSS 0 min, *n* = 3.

**Figure 4:**
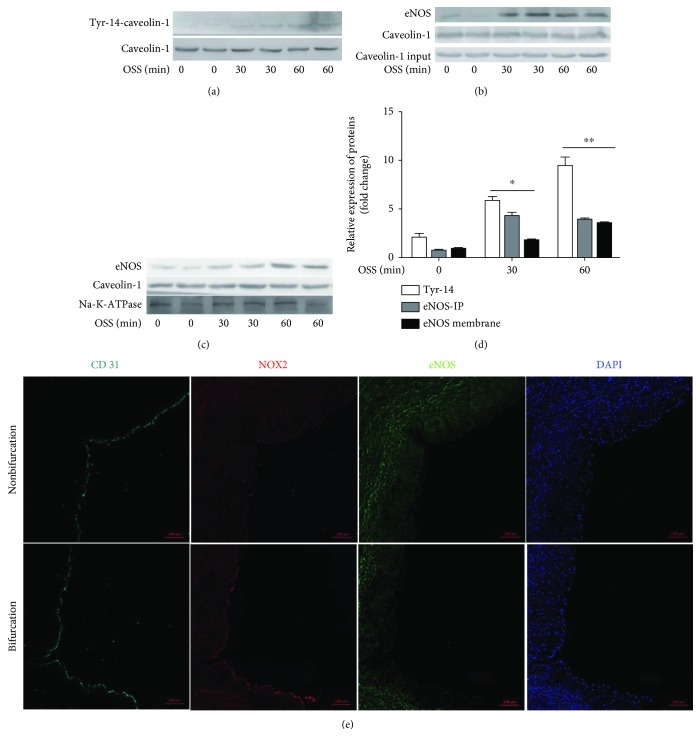
OSS suppresses eNOS. (a) OSS phosphorylates tyrosin-14 of caveolin-1. (b) Immunoprecipitation result shows increased association of eNOS and caveolin-1. (c) OSS promotes eNOS membrane location. (d) Bar diagram of the quantitative analysis of immunoblots. (e) Bifurcation and nonbifurcation arteries were stained with eNOS and NOX2. CD31 and DAPI were used to identify endothelial cells and nucleus, respectively. ^∗^*p* < 0.05 versus OSS 0 min, ^∗∗^*p* < 0.05 versus OSS 30 min, *n* = 3.

To explore the mechanisms in mediating OSS-induced oxidative stress, apocynin (10 *μ*mmol/L), allopurinol (1 mmol/L), tempol (1 mmol/L), and rotenone (100 *μ*mmol/L) were used prior to stimulation by OSS, followed by the examination of oxidative state. The flow cytometry results showed that the four inhibitors restored ROS accumulation, while apocynin, an NADPH oxidase inhibitor, being the most effective (Figures 3(a) and 3(b)). In ECs, activity of NADPH oxidase requires the assembly of NOX regulatory isoforms with its catalytic isoforms. The major NOX subunits in ECs are NOX1, NOX2, and NOX4. As shown by immunoblots, OSS significantly activated NOX2 and had no distinctive effect on NOX1 or NOX4 (Figures 3(c) and 3(d)). Moreover, OSS promoted translocation of p47 from cytoplasm to membrane and combination with NOX2 (Figures 3(e)–3(h)). To confirm NOX2 activation by OSS, we assayed its expression in human coronary artery. Compared to the nonbifurcation site, the bifurcation site had an increased expression of NOX2 (Figure 4(e)). The above results suggested that NOX2 plays an important role in OSS-induced redox reactions.

### 3.4. OSS Accelerates eNOS Membrane Localization

**Figure 5:**
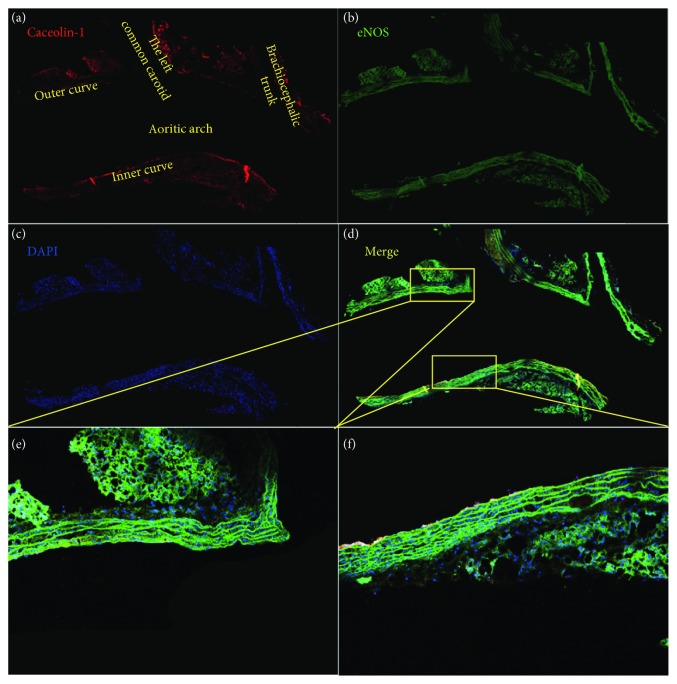
Colocalization of eNOS and caveolin-1 in the aortic arch. (a) Caveolin-1 and (b) eNOS were used to stain the aortic arch. (c) Nucleus was identified with DAPI. (d) A merged figure in which the outer and inner curves (the outer curve is where shear stress is high, and the inner curve is where shear stress is low and oscillatory) were enlarged.

NO derived from eNOS plays an important role in scavenging oxidant. In ECs, eNOS is functionally inhibited through the binding of caveolae-scaffolding domain. The phosphorylation of tyrosin-14, which is required for caveolae-mediated trafficking, showed a marked increase in OSS-treated cells (Figure 4(a)). In addition, OSS increased eNOS accumulation on membrane and promoted its colocalization with caveolin-1 (Figures 4(b)–4(d)). To verify their combination in vivo, we assayed the expression of eNOS and caveolin-1 in the aortic arch, where the outer curve is the normal stress region and the inner curve is the OSS region. Compared to the outer curve, an increased association of eNOS and caveolin-1 can be detected in the inner curve (Figures 5(a)–5(f)). The data identified that with OSS exposure, eNOS accumulated at membrane where it formed a complex with caveolin-1 and maintained inactive.

### 3.5. TLR4 Inhibition Deactivates NOX2 and Restores eNOS Activity

**Figure 6:**
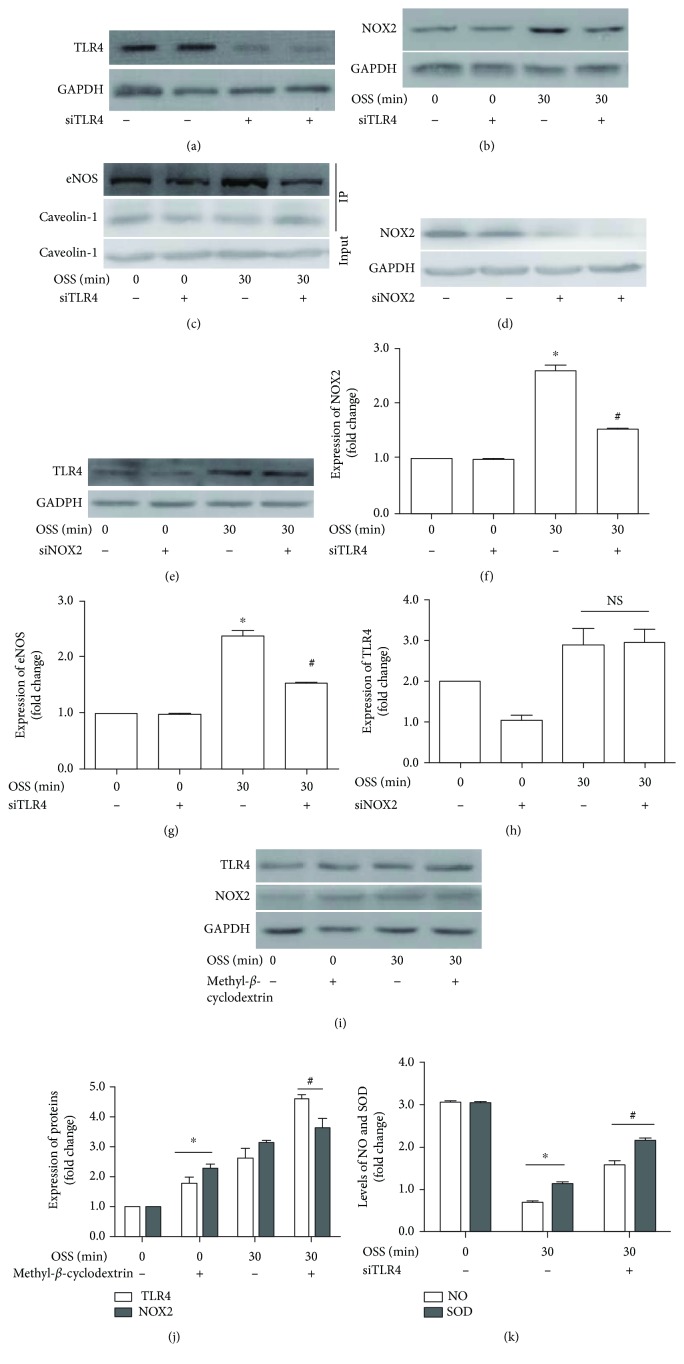
Interrelation among TLR4, NOX2, and caveolin-1 in OSS-treated cells. (a) Efficacy of siRNA against TLR4. (b) TLR4 inhibition decreases NOX2 expression with OSS exposure. (c) TLR4 inhibition suppresses the association of eNOS and caveolin-1. (d) Efficacy of siNOX2. (e) TLR4 level following NOX2 inhibition. (f–h) Quantitative analysis of immunoblots. (i) Expression of TLR4 and NOX2 with methyl-*β*-cyclodextrin treated or not. (j) Bar diagram showing quantitative data of immunoblots. (k) NO and SOD level in ECs with or without TLR4 inhibition under OSS conditions. ^∗^*p* < 0.05 versus OSS 0 min, ^#^*p* < 0.05 versus OSS 30 min without siTLR4 or methyl-*β*-cyclodextrin, *n* = 3.

**Figure 7:**
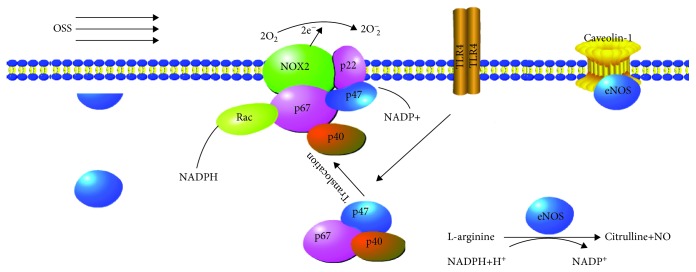
A schematic of how OSS promote oxidative stress through TLR4 activation. TLR4 modulates OSS-induced oxidative stress via two distinct mechanisms. First, it promotes the transfer of p47 from the cytoplasm to the cell membrane, activating NOX2. Second, it phosphorylates caveolin-1 and promotes the association of eNOS with caveolin-1, resulting in eNOS deactivation.

Since the TLR4 expression paralleled ROS generation, we posited that there might be bidirectional feedback between TLR4 activation and ROS production. Hence, we treated cells with siTLR4 or siNOX2 prior to OSS stimulation. We found that siTLR4 decreased the NOX2 expression and restored eNOS activity, whereas siNOX2 had no significant effect on TLR4 in sheared cells (Figures 6(a)–6(h)). Disrupting caveolae membrane domains with methyl-*β*-cyclodextrin (10 mmol/L) slightly increased TLR4 and NOX2 regardless of OSS treated or not (Figures 6(i) and 6(j)). Moreover, TLR4 inhibition restored NO production as well as SOD levels (Figure 6(k)). These results indicated that TLR4 acts as an upstream regulator of NOX2 and eNOS in OSS-induced oxidative damage (Figure 7).

## 4. Discussion

In the current study, we explored the mechanism of TLR4 activation in OSS-induced oxidative stress. The major findings were (1) OSS increased the TLR4 expression both in vivo and in vitro experiments, (2) TLR4 modulated OSS-induced oxidation by activating NOX2 and suppressing eNOS, and (3) TLR4 inhibition alleviated OSS-induced oxidative stress.

Atherosclerotic lesions are preferentially located at branch points and curved regions of the arterial tree, where blood flow is disturbed, suggesting that shear force contributes to the distribution of atherosclerotic lesions [3]. The application of shear stress to ECs can activate a number of mechanosensors that are associated with adaptor proteins and lead to modulation of signaling pathways [2, 4]. Emerging studies reveal that shear stress is converted into biochemical signals that are mediated by a variety of microdomains and membrane molecules, including caveolae, the glycocalyx, the cytoskeleton, ion channels, and G-protein-coupled receptors, followed by the almost simultaneous activation of multiple downstream signaling pathways [4]. TLRs, membrane-spanning proteins, have been implicated in the progression and development of many chronic diseases [15]. TLR4 has also been implicated in the development and progression of cardiovascular disease by inducing endothelial dysfunction [8, 10]. Study showed that TLR4 knockout led to marked reduction of aortic plaque area, decreased inflammation, and reduced oxidative damage [16]. In addition, TLR4 antagonist inhibited vascular inflammation and atherogenesis in ApoE^−/−^ mice [11]. Qu et al. reported that fibronectin containing the extra domain A (FN-EDA) was the activator of TLR4 under disturbed shear stress [17]. Together with our previous findings, we speculated that TLR4 could be a regulator of OSS-induced oxidative damage. In this study, we employed the CAC model and a parallel-plate flow chamber to mimic oscillatory flow change and assayed the TLR4 expression. Consistent with our hypothesis, cells in OSS regions showed a marked increase of the TLR4 expression both in vivo and in vitro study.

Oxidative stress is evoked immediately after imposing shear force [18]. In order to explore oxidative changes in response to TLR4 activation, we examined ROS accumulation in LPS-treated cells. LPS induced ROS production comparable with OSS exposure. Excess ROS can result in impairment of redox signaling which leads to cellular damage and dysfunction. An imbalance in the production of ROS and its breakdown can be both a cause and a consequence of oxidative stress [4, 19]. In the vasculature, several enzyme systems contribute to ROS formation, including NADPH oxidase, xanthine oxidase, cytochrome P-450 monooxygenases, mitochondrial oxidase, and uncoupled eNOS [20]. Although all of these enzymes contribute to oxidative burden, evidence is accumulating that NADPH oxidase acts as a major source of ROS [21, 22]. To verify this hypothesis, inhibitors of NADPH oxidase, xanthine oxidase, cytochrome P-450 monooxygenases, and mitochondrial oxidase were used prior to OSS exposure. As shown in our results, apocynin was the most efficient, suggesting that NADPH oxidase contributed to OSS-induced oxidative damage. NADPH oxidase consists of 7 catalytic subunits (NOX1-5, Duox1, and Duox2) and 5 regulatory subunits (p22, p47, p67, p40, and Rac). Of these, NOX1, NOX2, and NOX4 are expressed in cardiovascular ECs and participate in regulating endothelial function. In resting state, NOX2 binds with p22 and forms a membrane complex, while p67 links with p47 or p40 and thus forms a trimer in the cytosol. Upon stimulation, p47 transfers to membrane and binds with NOX2 to form an active enzyme [22]. Hence, we tested NOX1, 2, and 4 in the present study. OSS activated NOX2 and had no significant influence on NOX1 or NOX4. There are some controversy in shear stress-regulated NOXs. Hwang et al. reported activated NOX2 and NOX4 in ECs exposed to shear stress for 4 h [20]. Siu et al. have also reported that NOX2 is downregulated by LSS and NOX1 is actually affected by OSS [23]. The difference of flow pattern and stimulating time might contribute to these contradictory results. We think that the translocation of p47 from the cytoplasm to the membrane and its association with NOX2 may be the major source of redox activation under the acute stimulus of OSS.

Chen et al. reported that deactivated eNOS was accompanied by endothelial dysfunction in disturbed flow regions. eNOS serves as a critical enzyme in maintaining endothelial homeostasis, and the activation correlates with its intracellular localization [24]. In brief, eNOS is functionally inhibited through the binding of caveolae, a 50-100 nm diameter cell surface plasma membrane invagination, that plays a role in transcytosis, endocytosis, lipid homeostasis, and mechanotransduction [25, 26]. Caveolin-1 is the main functional protein of caveolae, and a loss of caveolin-1 results in the loss of caveolae. Direct binding of eNOS to caveolin-1 is a well-accepted mechanism for inactivating eNOS, while the absence of caveolin-1 is thought to promote eNOS dysfunction [27]. In this study, we assayed the eNOS membrane expression and its association with caveolin-1 to determine its activation state. Our study showed that OSS accelerated eNOS accumulation at the membrane, where it colocalized with caveolin-1 and resulted in deactivated eNOS. While disrupting caveolae with methyl-*β*-cyclodextrin excited TLR4 and NOX2 for the defection of caveolae usually leads to an extensively influence on cellular function, the regulation of caveolin-1 to TLR4 and NOX2 is controversial and needs to be explored further.

Several studies have reported the interrelation between TLR4 and NOXs. Wang et al. elaborated that TLR4 blocking reduced salt-induced prehypertension response via NADPH oxidases [28], while a study from Kim et al. also suggested a regulation effect of NOX4 on TLR4 [29]. We wondered the directional regulation; hence, siRNA against TLR4 or NOX2 were used prior to OSS exposure in ECs. TLR4 inhibition blocked NOX2 activation, while siNOX2 did not affect the TLR4 expression in sheared cells. In static-cultured cells, siNOX2 slightly decreased the TLR4 expression. Our data suggested that TLR4 acted as an upstream regulator of NOX2 in OSS-induced oxidative stress. Additionally, a bidirectional regulation existed between TLR4 and NOX2 in static-cultured cells which need further exploration.

There are several limitations to our study. In vivo study, a constriction cast was used to modify shear stress in carotid; however, we cannot exclude the injury caused by the constricted surgery; hence, a nonconstricting cast will be better used as sham. In the physiological state, the vessel wall is exposed to multiple stimuli in addition to shear stress; hence, potential contribution of other stimuli such as stretch force or chemical irritants is difficult to study. In addition, endothelial dysfunction is a consequence of chronic and complex stimuli and cannot be fully explained by acute stimulus; thus, a prolonged time course study should be conducted in the future. Moreover, classical TLR activation contributes to immune regulation through macrophages instate of mediated oxidative stress in ECs. Therefore, dual directional regulation of macrophages and ECs in a coculture system should be further explored.

In conclusion, our study identifies the specific roles that TLR4 plays in the response of endothelial cells to OSS. Under OSS condition, TLR4 promotes the translocation of p47 from cytoplasm to the membrane and combination with NOX2 which results in the production of O2-. On the other hand, OSS triggers the colocalization of eNOS with caveolin-1 which in turn deactivates eNOS and inhibits the production of NO. These results not only elucidate the key details of OSS regulation of endothelial redox status but also identify possible therapeutic targets that can be exploited in the treatment of endothelial injury that are high prevalent in vasculature exposed to oscillatory flow.

## Data Availability

The data including ultrasound testing data, immunochemistry data, western blot data and biochemistry data used to support the findings of the study are available from the corresponding author upon request.
